# Genes of susceptibility to early neurodegenerative changes in the rat retina and brain: analysis by means of congenic strains

**DOI:** 10.1186/s12863-016-0461-7

**Published:** 2016-12-22

**Authors:** Elena E. Korbolina, Anna A. Zhdankina, Anzhela Zh. Fursova, Oyuna S. Kozhevnikova, Natalia G. Kolosova

**Affiliations:** 1 Institute of Cytology and Genetics, SB RAS, Novosibirsk, Russia; 2 Siberian State Medical University, Tomsk, Russia; 3 Novosibirsk State University, Novosibirsk, Russia

**Keywords:** Genetic architecture of complex trait, OXYS rats, Quantitative trait locus, Congenic strain, Alzheimer’s disease, Age-related macular degeneration

## Abstract

**Background:**

There has been considerable interest in discovery of the genetic architecture of complex traits, particularly age-related neurodegenerative disorders. To predict disease risk and to understand its genetic basis in humans, it is necessary to study animal models. Our previous research on the accelerated-senescence OXYS strain has revealed two quantitative trait loci (QTLs) on rat chromosome 1 that are associated with early cataract and/or retinopathy as well as with behavioral abnormalities. Each locus was partially mapped within the introgressed segments in a certain congenic strain: WAG/OXYS-1.1 or WAG/OXYS-1.2. Retinal transcriptome profiling of 20-day-old congenic and OXYS rats by high-throughput RNA sequencing uncovered relevant candidate genes and pathways. Nonetheless, the question remained open whether the same genetic components simultaneously have effects on various manifestations of the accelerated-senescence phenotype in OXYS rats. The present study was designed to analyze the genes of susceptibility to early neurodegenerative processes taking place in the OXYS rat retina and brain and to assess their potential functional clustering. The study was based on the findings from recent publications (including mapping of quantitative trait loci) and on comparative phenotyping of congenic rat strains.

**Results:**

The backcrossing of Wistar Albino Glaxo (WAG) and OXYS strains to generate the congenics resulted in two congenic strains with high susceptibility to cataract and retinopathy but with no obvious signs of Alzheimer’s disease-like brain pathology that are specific for OXYS rats. Thus, the genes of susceptibility to brain neurodegeneration were not introgressed into the congenic strains or there is a strong effect of the genetic background on the disease phenotype. Moreover, the progression of retinopathy with age was relatively less severe in the WAG background compared to the OXYS background. A comparative analysis of previously defined QTLs and congenic segments led to identification of candidate genes with a suspected effect on brain neurodegeneration including the genes showing differential expression in the congenic strains.

**Conclusion:**

Overall, our findings suggest that the cause of the cataract and the cause of retinopathy phenotypes in OXYS rats may be genetically linked to each other within the introgressed segments in the WAG/OXYS-1.1 and/or WAG/OXYS-1.2 congenic strains.

**Electronic supplementary material:**

The online version of this article (doi:10.1186/s12863-016-0461-7) contains supplementary material, which is available to authorized users.

## Background

The lifespan and health-span are intricately entwined complex traits influenced by many genes that can either predispose to age-related diseases and accelerate aging or slow the aging process [1, 2]. In recent years, large-scale genetic studies advanced our understanding of the genetic architecture of many complex heritable traits. The term “genetic architecture” can be directly described for any trait based on information regarding gene and allele number, the distribution of allelic effects, and patterns of their relations. The most common age-related disorders in humans are heritable, meaning that a part of the phenotypic variation is defined by the variation in genetic components. Numerous findings reveal that the molecular genetic mechanisms may have much in common, particularly for age-related neurodegenerative disorders, including the most common senile dementia, Alzheimer’s disease (AD), and age-related macular degeneration (AMD), which is the leading cause of visual impairment and blindness in the developed countries [3–7]. Nevertheless, the complex underlying heredity in most cases of AMD and AD remains elusive primarily due to the lack of reliable samples at different stages of the disease. Postmortem samples of brain or eye tissues can be used only to observe the end results of neurodegenerative processes, which do not necessarily reflect the mechanisms responsible for disease development. Thus, much of research has been traditionally based on the use of animal models, to both improve our understanding of the pathophysiological mechanisms of the disease and to test novel therapeutic approaches [8–12]. We believe that the use of accelerated-senescence OXYS rats, which spontaneously develop both AMD-like retinopathy and the key signs of Alzheimer’s disease, represent unique opportunities for research in this field.

The OXYS strain was derived from outbred Wistar stock at the Institute of Cytology and Genetics by selection and inbreeding of Wistar rats that were sensitive to the cataractogenic effects of galactose-enriched diet, as described earlier [13, 14]. The development of cataracts was induced in Wistar rats by galactose overconsumption in the first five generations of inbreeding. Subsequently, only the rats with early spontaneous cataract were selected and bred using brother-sister inbreeding to generate the accelerated-senescence OXYS strain in the 1970s. We have demonstrated that OXYS rats inherit the characteristic accelerated-senescence phenotype in a linked manner, including sarcopenia [15], osteoporosis [16], AMD-like retinopathy [17, 18], and AD-like pathology [13, 19]. These findings led to the use of OXYS strain in the relevant basic research and in the studies on therapeutic effectiveness of various drugs [20–22]. We assumed that mutations affecting the development of early cataract in senescence-accelerated OXYS rats also have effects on early manifestation of other age-related disorders. Using this strategy, we identified two quantitative trait loci (QTLs) on rat chromosome 1 that are associated with early cataract and/or AMD-like retinopathy and with certain behavioral signs of brain neurodegeneration in OXYS rats. The candidate QTLs named QTL1 and QTL2 are located in the regions of the microsatellite markers D1Rat30-D1Rat219 and D1Rat219-D1Rat81, respectively [23]. To confirm the findings, the segments of chromosome 1 of OXYS rats within QTL1 and QTL2 were introgressed in interval-specific congenic strains. The parental strains were accelerated-senescence strain OXYS (the donor of segments of chromosome 1) and the inbred Wistar Albino Glaxo (WAG) rat strain (as a recipient with a normal phenotype). The rats selected from the 8^th^ generation of backcrossing to the WAG strain (Additional file 1: Figure S1) were mated to obtain the introgressed segments in the homozygous state. The result was WAG.OXYS-(*D1Rat30-D1Rat219*) and WAG.OXYS-(*D1Rat219-D1Rat81*) rat congenic strains hereafter referred to as WAG/OXYS-1.1 and WAG/OXYS-1.2, respectively. Examination of the retinal transcriptome at 20 days of age identified a number of candidate genes and functionally enriched pathways of differentially expressed genes in WAG/OXYS-1.1 and WAG/OXYS-1.2 congenic strains when compared with the OXYS parental strain. Nevertheless, mapping by means of RNA-seq data showed that the QTL-derived segments of OXYS chromosome 1 were only partially introgressed in the resulting WAG/OXYS-1.1 and WAG/OXYS-1.2 congenic strains [24]. The ultimate aim of the present study was to gain a better understanding of the genes of susceptibility to neurodegenerative changes in the OXYS retina and brain. To test our previous QTL-derived hypothesis further, we carried out comprehensive phenotyping of WAG/OXYS-1.1 and WAG/OXYS-1.2 congenic rat strains in comparison with the parental strains. The advantages of the two congenic rat strains allowed us to analyze the RNO1 candidate genes with a specific emphasis on phenotypic variation.

## Results

### Analysis of phenotypic traits in WAG/OXYS-1.1 and WAG/OXYS-1.2 congenic strains

#### Cataract and retinopathy progression with age

OXYS rats develop an early-onset ocular pathology characterized by cataract and retinopathy, and the cataract severity considerably progresses with age against the background of neurodegenerative alterations in the OXYS retina. To test whether the congenic WAG/OXYS strains develop a similar pathology, we analyzed the progression of cataract and retinopathy in the experimental groups (*n* = 14 to 15) of the parental (OXYS and WAG) and congenic (WAG/OXYS-1.1 and WAG/OXYS-1.2) rat strains between the ages of 2 and 12 months.

**Fig. 1:**
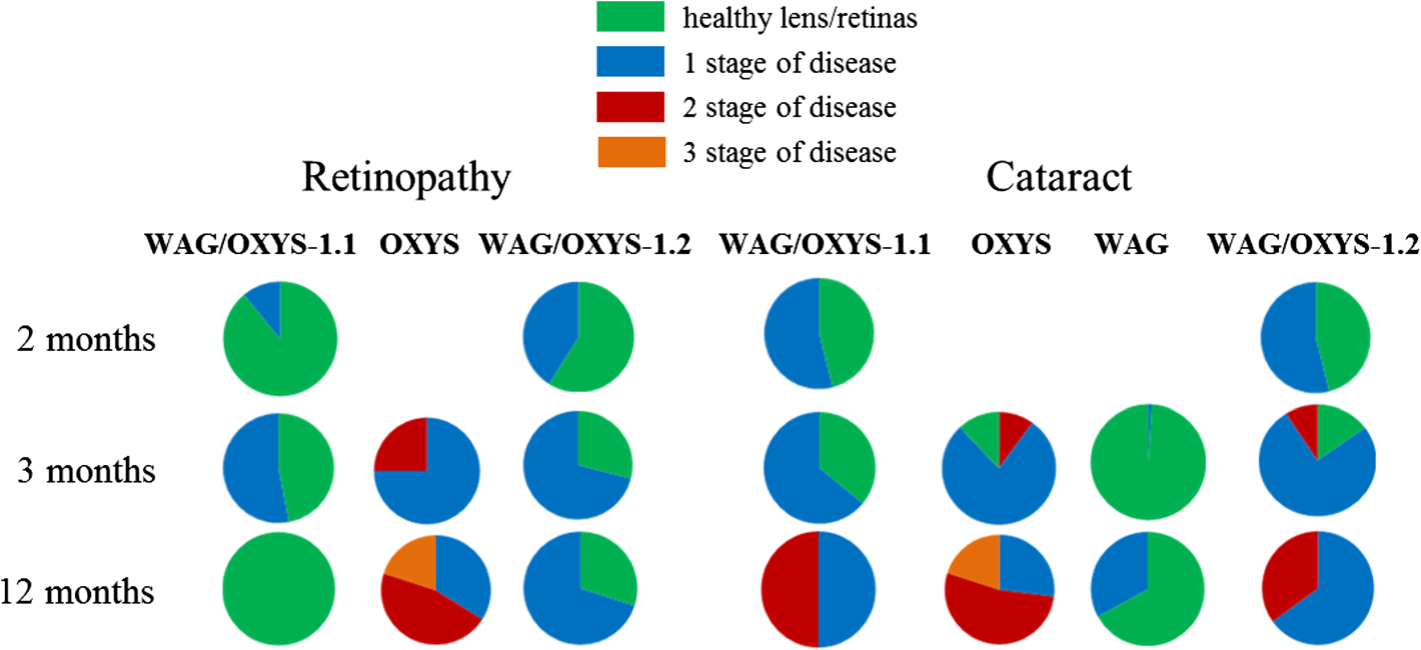
The incidence of cataract and retinopathy in congenic (WAG/OXYS-1.1 and WAG/OXYS-1.2) and parental (OXYS and WAG) rat strains at the age of 2, 3, and 12 months. We did not observe any cases of retinopathy in WAG rats at any age. Data are presented as a distribution of rat eyes with the stages of cataract and retinopathy (*n* = 30 eyes). Colors are labeled with 0 through 2 and correspond to a certain stage of cataract or retinopathy, scoring 0–3, as explained in Methods

At 3 months of age, the clinical signs of cataract and retinopathy were observed in 89% of lenses and in 100% of retinas in OXYS rats (*n* = 30, Fig. 1); 9% of eyes in the 3-month-old OXYS group matched the 2^nd^ stage of cataract and 25% of eyes corresponded to the 2^nd^ stage of retinopathy according to the AREDS protocol [25]. The severity of both cataracts and retinopathy progressed dramatically with age in OXYS rats in agreement with our prior data [17, 26, 27]. By 12 months of age, 27% of OXYS lenses (*n* = 30) corresponded to the 1^st^ stage, 53% of lenses to the 2^nd^ stage, and 20% of lenses to the 3^rd^ stage of cataract; the last did not allow us to evaluate the state of ocular fundus. In 58% of 12-month-old OXYS rat’s eyes, for which the evaluation was still possible, the pathological changes of the retina corresponded to the 2^nd^ stage of disease. For comparison, only a single case of the 1^st^ stage cataract and no cases of retinopathy were registered within the WAG experimental group [23].

The progression of cataract and retinopathy in WAG/OXYS-1.1 and WAG/OXYS-1.2 rat strains differs clinically from both parental strains (Fig. 1). In 3-month-old WAG/OXYS-1.2 rats, the signs of the 1^st^ stage of cataract and 1^st^ stage of retinopathy were observed in 71% of eyes (*n* = 30) with no signs of progression to the 2^nd^ stage for both diseases. At the age of 12 months, 100% of WAG/OXYS-1.2 rats were characterized by lens alterations, and 35% of WAG/OXYS-1.2 lenses corresponded to the 2^nd^ stage of cataract. We identified the signs corresponding to the 1^st^ stage of retinopathy in 70% of the eyes in 12-month-old WAG/OXYS-1.2 rats. We similarly did not observe any signs of the 2^nd^ stage of retinopathy even in 12-month-old animals.

In the group of 3-month-old WAG/OXYS-1.1 congenic rats, the signs of the 1^st^ stage of cataract were observed in 64% of lenses (*n* = 30), with no signs of progression to the 2^nd^ stage of cataract. By the age of 12 months, the incidence of cataracts had reached 100% as in WAG/OXYS-1.2 rats. At 12 months of age, 50% of WAG/OXYS-1.1 lenses showed the 1^st^ stage of cataract, and the other 50% of lenses the 2^nd^. At the age of 3 months, we observed the signs of the 1^st^ stage of retinopathy in 53% of eyes of WAG/OXYS-1.1 rats, with no cases of the 2^nd^ stage. The number of affected animals and eyes did not increase with age in WAG/OXYS-1.1 rats. Moreover, ophthalmoscopic examination of the same animals at the age of 12 months revealed 100% remission of retinopathy, just as in the results of periodic ophthalmic examinations during the breeding WAG/OXYS-1.2 rats. The remission has never been observed in OXYS rats except in the experiments involving a therapeutic intervention.

In sum, our findings indicate that WAG/OXYS-1.1 and WAG/OXYS-1.2 rats develop cataracts and retinopathy, but the progression of both diseases was relatively less severe in the WAG background compared to the OXYS background even at 12 months of age.

### Histological examination of rat retinas

**Fig. 2:**
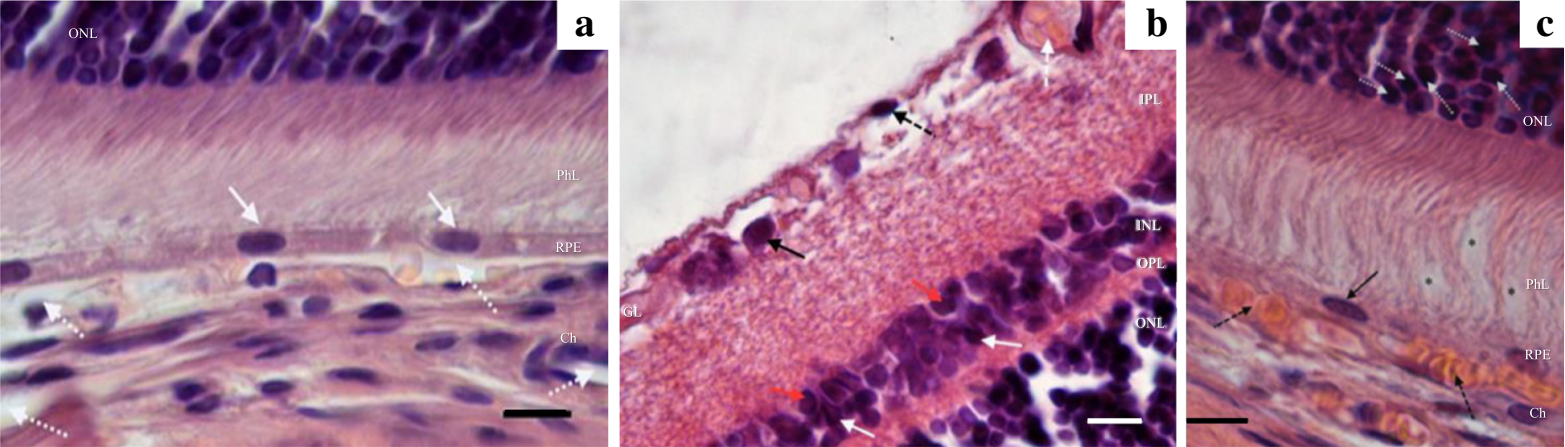
Retinas of 3-month-old WAG **a** and OXYS **b c** rats. **a** Open chorioretinal vessels with a small amount of blood cells (*white dotted arrows*); the cellular prismatic pigment epithelium with prominent microvilli (*white arrows*). **b** The ganglion neuron nucleus with pyknosis (*black arrow*); hyperchromic oligodendrocyte with nuclear pyknosis in the layer of nerve fibers (*dashed black arrow*); stasis of blood cells in the intraretinal vessel (*white dashed arrow*); hyperchromic pyknomorphic radial glial cells (*white arrows*); hyperchromic pyknomorphic associative neurons (*red arrows*). **c** Stasis and sludge of the blood cells in the choroidal vessels (*black dashed arrows*); a pyknotic nucleus of an RPE cell (*black arrows*); disorientation and disaggregation of the outer segments of photoreceptors (*asterisk*); the photoreceptors containing a nucleus with pyknosis (*white dotted arrows*). Abbreviations: OPL, outer plexiform layer; INL, inner nuclear layer; IPL, the inner plexiform layer; ONL, outer nuclear layer; GL, ganglion cell layer, RPE, retinal pigment epithelium, Ch, choroid. The scale bar is 10 μm

At the next step, we compared the histological features of the retina in the congenic (WAG/OXYS-1.1 and WAG/OXYS-1.2) and parental (OXYS and WAG) rat strains at the age of 3, 10, and 16 months. As we reported earlier (data not shown), the development of retinopathy in OXYS rats is accompanied by progressing degenerative alterations that primary affect the RPE cells and lead to choriocapillaris atrophy and a complete loss of photoreceptor cells in the rats’ retina by the age of 24 months [17]. According the data of the present study, at the age of 3 months, there were prominent abnormalities of the choroidal vasculature, RPE cells, and neurosensory cells in the retina of OXYS rats, while the retina of WAG rats showed normal structure. Unlike the choroid of WAG rats (Fig. 2a), the choroid of OXYS rats was characterized by incident aggregation of blood cells, stasis, and thrombosis of small vessels. The specific area of vessels with signs of partial occlusion was significantly greater in OXYS rats as well (Fig. 2b). In the photoreceptor cells of OXYS rats, there were nuclear pyknosis (the clumped chromatin) and disaggregation and disorientation of membrane discs of outer segments (Fig. 2c). In the inner retinal layers of OXYS rats, we observed hyperchromatic pyknomorphic neurons and swollen neurons in association with radial glial cells, which were hyperchromic and pyknomorphic. In the ganglionar layer of OXYS rats’ retina, some neurons were pyknomorphic and chromolytic as well, indicative of a decline in the reserve capacity of neurons and a characteristic sign of hypoxia. The progression of these abnormalities in OXYS rats to the age of 10 months was accompanied by a significant reduction in thickness of the photoreceptor cell layer and a reduction in the number of photoreceptor cell nuclei of the outer nuclear layer, especially in the central part of the retina, while in the WAG rats the retina remained normal.

**Fig. 3:**
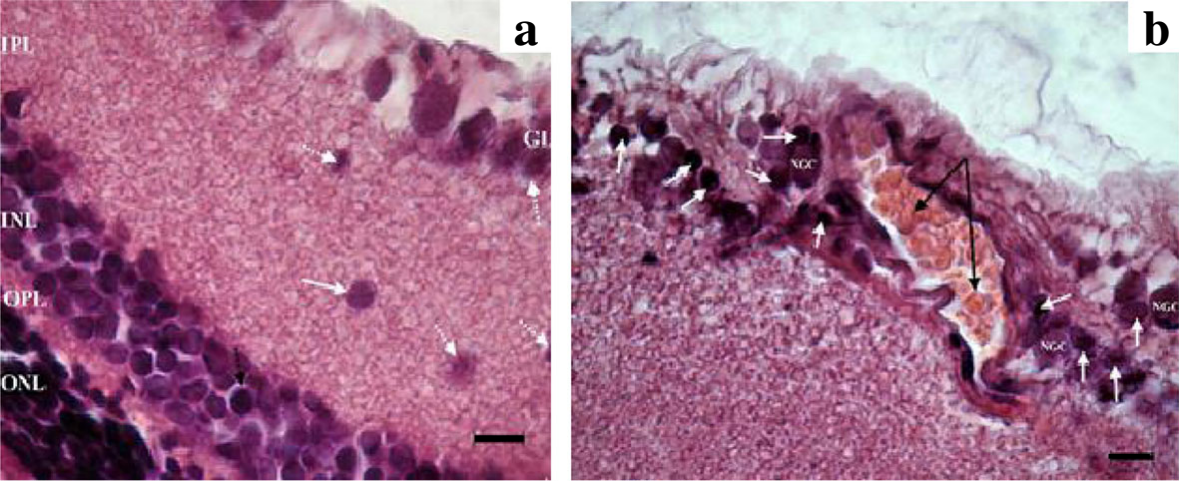
Retinas of 3-month-old WAG/OXYS-1.2 rats. **a** Migration of macrophages into the inner plexiform and ganglion layers (*white arrows*). Migration of the perikaryon of the associative neuron into the inner plexiform layer (*dashed black arrow*). **b** Advanced intraretinal capillary with phenomenon of the blood cells stasis (black arrows); lymphocytic infiltration (*white arrows*); NGC: the nucleus of the ganglion neuron. Abbreviations: OPL: outer plexiform layer; INL: inner nuclear layer; IPL: the inner plexiform layer; ONL: outer nuclear layer; GL: ganglion cell layer. The scale bar is 10 μm

Some degenerative changes were observed in the retina of 3-month-old WAG/OXYS-1.1 and WAG/OXYS-1.2 congenic rats. In contrast to OXYS rats, these alterations developed against the evident inflammatory background, including the mass migration of lymphocytes and macrophages into the inner plexiform layer and ganglion cell layer. Such a pattern is typical for AMD patients, but not for the OXYS parental strain [28]. At the same time, the predominantly damaged retinal layers in congenic rats were the ganglion cell layer and the inner nuclear layer. Disturbances of microcirculation were identified such as the phenomena of blood cell stasis and thrombosis of inner retinal vessels of the WAG/OXYS-1.1 and WAG/OXYS-1.2 retinas (Fig. 3). Histopathological features of retinal alterations in 10-month-old congenic rats were generally similar to those observed in age-matched OXYS rats. Furthermore, the massive accumulation of lipofuscin granules was detected in the RPE cells of OXYS rats, but not of congenic rats. The functionally active open choroid capillaries with a few blood cells were observed in the retina of WAG/OXYS-1.1 and in the retina of WAG/OXYS-1.2 rats. Nevertheless, the specific area of choroid vessels was significantly reduced in the 10-month-old WAG/OXYS-1.1 rats when compared to age-matched OXYS and WAG/OXYS-1.2 rats (*p* = 0.007 for pairwise comparisons). At this age, a significantly increased area of inner retinal vessels was observed in the retina of rats of both congenic strains when compared to OXYS rats (*p* < 0.05).

**Fig. 4:**
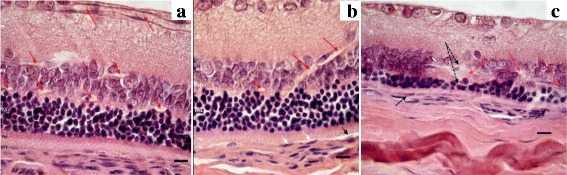
Retinas of 10-month-old WAG/OXYS-1.2 **a** and WAG/OXYS-1.1 **b** rats. The thinning of the outer nuclear layer with a reduction in the number of the photoreceptors in the retina of a 16-month-old WAG/OXYS-1.2 rat **c. a** The open intraretinal vessels (*red arrows*). **b** Open intraretinal capillaries (*red arrows*); the flattening of the RPE cells, RPE cells containing a nucleus with pyknosis (*white dashed arrows*); migration of mononuclear phagocytes within the subretinal space with RPE detachment (*black dashed arrow*). **c** Open intraretinal capillaries (*red arrows*); the flattening of the RPE cells, RPE cells containing a nucleus with pyknosis (*black arrows*); the destructive changes in neurosensory and associative neurons (*black dashed arrows*). Abbreviations: OPL: outer plexiform layer, INL: inner nuclear layer; IPL: the inner plexiform layer; ONL: outer nuclear layer; GL: ganglion cell layer, RPE: retinal pigment epithelium, PhL: photoreceptor layer. The scale bar is 10 μm

Photoreceptors with the signs of pyknosis as well as the migration of some photoreceptor nuclei to the outer plexiform and the inner nuclear layers were detected in the retinas of both WAG/OXYS-1.1 and WAG/OXYS-1.2 congenic rats. The qualitative evaluation revealed that the number of rows of photoreceptor nuclei was greater in the congenic rats than in age-matched OXYS rats even at age 10 months. A comparative reduction in the number of photoreceptor nuclei of the outer nuclear layer in WAG/OXYS-1.1 and WAG/OXYS-1.2 rats with respect to OXYS rats was observed by the age of 16 months during partial occlusion of choroidal vessels and flattening of the RPE cells (Fig. 4).

In sum, the histopathological assessment of rat retinas confirmed the presence of pathological changes in OXYS, WAG/OXYS-1.1, and WAG/OXYS-1.2 rats (but not in WAG rats) as early as age 3 months. The retinopathy features including the predominantly damaged retinal layers differ between the congenic strains and OXYS strain but seem generally similar between WAG/OXYS-1.1 and WAG/OXYS-1.2 strains. By the age of 16 months, we detected major differences between the congenic and OXYS rats in the retinal blood supply (Additional file 1: Figure S1).

### Evaluation of neurodegenerative brain alterations

#### Behavioral testing

In the present study, 3-month-old OXYS rats demonstrated considerably reduced locomotor activity in the open field (OF) test as compared to age-matched WAG controls: the number of crossed squares was significantly lower in OXYS rats (*p* <0.05). According to pairwise comparisons no significant differences were detected in the locomotor activity between WAG/OXYS-1.1 and OXYS rats. Among WAG/OXYS-1.2 rats, the number of crossed squares in the OF test varied considerably and was significantly greater than that in OXYS rats (*p* = 0.0001), likely indicating a reduced level of anxiety (Additional file 1: Figure S2). In the elevated plus-maze (EPM) test, the rats of both congenic strains did not differ from either WAG or OXYS rats in the level of locomotor activity and anxiety (analyzed by means of the number of entries into the open arms and the time spent in the open arms).

**Fig. 5:**
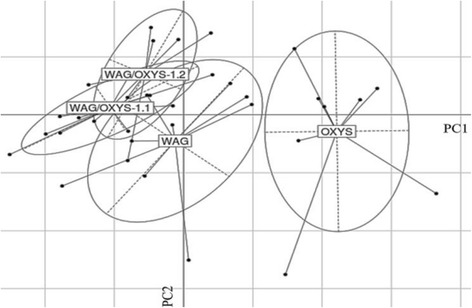
Results for the two-dimensional principal component analysis (PCA) of rat behavioral parameters recorded in the 8-arm radial maze test. According to the results of PCA, the rats of two congenic strains, WAG/OXYS-1.1 and WAG/OXYS-1.2, proved to be much closer to the parameters of WAG rats, than to those of OXYS rats. Legend: PC1 and PC2: principal components 1 and 2, respectively

In order to estimate the reference memory, the learning ability of 5-month-old OXYS, WAG, and congenic rats (*n* = 7 to 8 per experimental group) was assessed in the 8-arm radial maze. Performance of working and reference memory was combined for four training periods according the standard test variation: the 1^st^ training period, day 1; the 2^nd^, days 2–4; the 3^rd^, days 5–7; and the last training period, days 8–10 of training. The number of total arm entries (used to estimate a rat’s horizontal locomotor activity) of OXYS rats in the 8-arm radial maze was significantly lower, and the number of grooming events (used to estimate animal’s anxiety) was higher than in WAG and both congenic strains (*p* <0.05). WAG/OXYS-1.1 rats also showed a tendency for an increase in the number of grooming acts in the 2^nd^ training period (*p* = 0.645). No significant differences in the number of working memory errors (the number of re-entries into already visited arms of the maze during testing) and memory error rates (the ratio of the number of entries into the arms nonreinforced with a food stimulus to the total number of entries during test sessions, expressed in percentage points) for all training periods were demonstrated for the congenic and parental WAG experimental groups. Nonetheless, the Monte Carlo sampling of the recorded parameters revealed significant interstrain differences between all experimental groups: OXYS, WAG, WAG/OXYS-1.1, and WAG/OXYS-1.2 rats (*p* <0.01 for each pairwise comparison). Fig. 5 depicts the results of principal component analysis (PCA) of combined rat behavioral parameters in the 8-arm radial maze.

#### Morphometric analysis of the rat brain

**Fig. 6:**
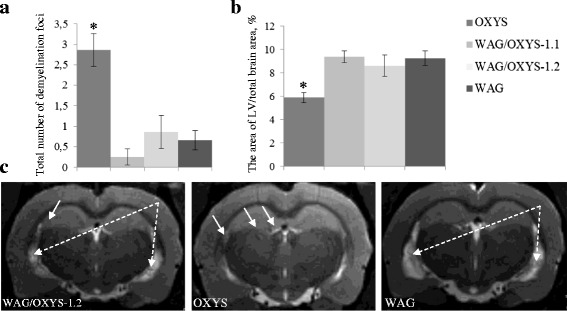
An MRI morphometric study of the brain of 12-month-old OXYS, WAG/OXYS 1.1, and WAG/OXYS-1.2 rats. **a** The total number of demyelinating foci is significantly greater in the OXYS brain than in the brain of the congenic and WAG rats. **b** The lateral ventricles in the congenic and WAG rats are enlarged when compared with OXYS rats. **c** Axial slices of the brain of 12-month-old WAG/OXYS-1.2, WAG, and OXYS rats. The foci of demyelination (white arrows) and the increase in the size of lateral ventricles in WAG and congenic rats (white dotted arrows) are visible. Not all congenic and WAG rats have the demyelination lesions. The data are shown as mean ± SEM. Abbreviation: LV, lateral ventricles; *P < 0.05 for differences between the strains

According to the MRI analysis, the rats of both congenic strains developed some signs of neurodegenerative alterations by 12 months of age, but those were found to be less pronounced than in 12-month-old parental OXYS rats. Demyelination is the process where the myelin sheath of neuronal cells is damaged. At the age of 12 months, the demyelination lesions were detected in the brains of 100% of OXYS rats, 32% of WAG rats, 25% of WAG/OXYS-1.1 rats, and 43% of WAG/OXYS-1.2 rats. At the same time, the foci of demyelination were observed generally in the corpus callosum of WAG/OXYS-1.1 rats and in the hippocampus of OXYS and WAG/OXYS-1.2 rats (the total number of demyelination lesions is given in Fig. 6a). The total number of demyelination lesions in T2-weighted images was orders of magnitude greater in the corpus callosum of OXYS rats than in either congenic strain (*p* <0.001) and twice larger in the hippocampus of OXYS rats than in the hippocampus of the WAG/OXYS-1.2 congenic strain (*p* <0.091, a nonsignificant tendency). Enlargement of the lateral ventricles, called ventriculomegaly, is associated with neurodegenerative diseases, such as Alzheimer’s disease, while the initial cause is often unknown. This could be due to an imbalance between production and resorption of cerebrospinal fluid, typical for neurodegenerative diseases [29, 30]. According to the data, the specific area of the cerebral lateral ventricles was increased in WAG and both congenic strains when compared with OXYS rats (*p* <0.001) (Fig. 6b, c). Thus, the present study confirmed the presence of previously described neurodegenerative changes in the brain of OXYS rats, progressing dramatically with age [13]. Nonetheless, there were no significant differences between rats of congenic (WAG/OXYS-1.1 and WAG/OXYS-1.2) and WAG strain in terms of morphological and functional brain parameters.

### Comparison of the results on candidate genetic loci mapping on rat chromosome 1

Candidate QTL1 and QTL2 loci on rat chromosome 1 were associated with early cataract and retinopathy development as well as with some characteristic behavioral parameters of OXYS rats [23]. The effects on cataract and retinopathy were confirmed in WAG/OXYS-1.1 and WAG/OXYS-1.2 congenic strains. In the present study, we showed convincingly that animals carrying OXYS alleles at each of congenic loci were not susceptible to development of brain lesions when compared with the WAG parental strain. Thus, we could reasonably assume that in the congenic segments, there was likely no gene or gene cluster whose structural and/or functional defects led to cerebral pathophysiological processes.

**Fig. 7:**
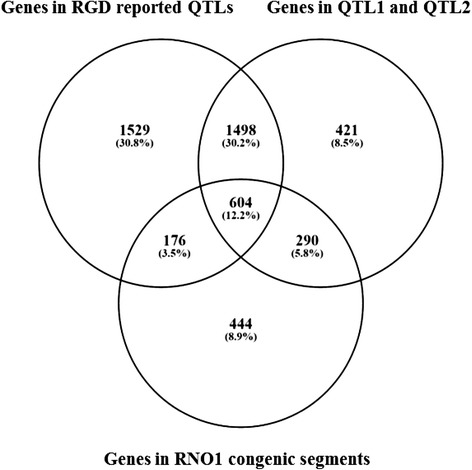
The Venn diagram showing overlaps among the three analyzed sets of RNO1 genes. The genes located within the previously determined QTL1 and QTL2 [23], the genes located within the mapped congenic segments in WAG/OXYS-1.1 and WAG/OXYS-1.2 rat congenic strains [24], and the genes located within rat QTLs, presented in RGD were analyzed. The number of genes in each group as reported by RGD is shown in the corresponding field

Assuming that the point of largest linkage was estimated in the 95% confidence interval, we produced a complete list of genes within each of the two previously determined QTLs (Additional file 2: Table S1). According to our previous RNA-seq-based analyses, the candidate QTL1 and QTL2 loci do not exactly match the congenic segments: the regions of OXYS chromosome 1 that were introgressed into WAG/OXYS-1.1 or WAG/OXYS-1.2 congenic strains. Therefore, we compared the list of genes with a) the list of genes located within the mapped congenic segments and b) with those genes that were identified within rat QTL loci associated with behavioral abnormalities according to the Rat Genome Database [31]. The Venn diagram in Fig. 7 shows the relations among the three overlapping sets of genes (Additional file 2: Tables S2–S5). In particular, we found that genes involved in the synaptic transmission, in the endoplasmic reticulum membrane, cell adhesion, protein complex biogenesis and, in particular, the ribonucleoprotein complex are represented in the intersection of 1498 genes, which fall into both of RGD QTL and QTL1/QTL2 regions but do not fall into congenic segments (Additional file 3). Table 1 shows the top enriched GO categories in this set of 1498 genes. Some of the identified genes were particularly relevant to the neurological phenotype, including 11 genes of Alzheimer’s disease-presenilin pathway [Panther: P00004], and 8 genes of Alzheimer’s disease-amyloid secretase pathway [Panther: P00003].

**Table 1:** A DAVID report on top representative enriched GO categories for the set of 1498 analyzed RNO1 genes. Some broad categories associated with sensory perception and olfaction were excluded

Category	Term	Count	*P* Value
GOTERM_BP_FAT	GO:0050877, neurological system process	210	5.847029238110996E-27
	GO:0050890, cognition	192	7.182253845261601E-27
	GO:0007186, G-protein coupled receptor protein signaling pathway	202	4.097419923337612E-18
	GO:0007166, cell surface receptor linked signal transduction	222	6.215501968023257E-13
	GO:0006414, translational elongation	15	7.078201738398857E-4
	GO:0015671, oxygen transport	5	0.0032335241561143833
	GO:0051259, protein oligomerization	22	0.00669002198721339
	GO:0006836, neurotransmitter transport	13	0.009111208533119846
	GO:0006974, response to DNA damage stimulus	24	0.01657678971411233
	GO:0007268, synaptic transmission	21	0.019269327596189923
	GO:0033554, cellular response to stress	34	0.04221800073686602
	GO:0001505, regulation of neurotransmitter levels	10	0.042368672097131105
	GO:0006261, DNA-dependent DNA replication	6	0.04679384686345697
GOTERM_CC_FAT	GO:0031224, intrinsic to membrane	323	1.0902250124386092E-7
	GO:0033279, ribosomal subunit	12	0.008194644308006482
	GO:0045211, postsynaptic membrane	16	0.013015080960369218
	GO:0045202, synapse	33	0.020591521818732007
	GO:0022626, cytosolic ribosome	8	0.02681660832216637
	GO:0030894, replisome; GO:0043596, nuclear replication fork	4	0.03589946869050226

Count: the number of genes in a category; the *P* values were given by DAVID

After that, we compared the same list of 1498 genes with the list of genes for which the differential expression was reported previously [24] in WAG/OXYS-1.1 or WAG/OXYS-1.2 retinas when compared with the OXYS strain at 20 days of age (Additional file 4). Only reads uniquely mapped to the reference genome were analyzed at the cutoff of |log2FC| ≥ 1.0; padj < 0.1 (where FC is fold change) for the WAG/OXYS-1.1 versus OXYS and WAG/OXYS-1.2 versus OXYS differentially expressed (DE) genes. We found that the level mRNA of the *Asrgl1* gene, also known as *Gliap*, was significantly higher in the retina of both congenic rats, than in the retina of OXYS rats (adjp-value 0.0342 and 0.0000013 for WAG/OXYS-1.1 and WAG/OXYS-1.2 strains, respectively). The level of mRNA for two genes among 1498, *Dhdh*, and *Gpha2* (located within previously reported QTL1 and QTL2, respectively) was significantly lower in the retina of WAG/OXYS-1.2 rats than in the retina of OXYS rats (adjp-value 0.0863 and 0.0561, respectively). Thus, our data are suggestive of a differential effect of the genetic background on regulation of the *Asrgl1*, *Dhdh*, and *Gpha2* genes.

In addition, the chromosomal regions delineated in the intersection of the *Arrd2* locus, QTL for age-related retinal degeneration, and the congenic segment introgressed into WAG/OXYS-1.2 strain contain the potential candidate gene *Fxn*, frataxin [RGD: 1565754], which was shown to be associated with mitochondrial abnormalities, neurite degeneration, and apoptotic cell death.

## Discussion

Studying ageing is a challenging task because of the complexity of the aging process and the asynchrony of senescence that was documented in humans, laboratory models, and wild animals [32–34] as well as among phenotypic traits of a natural population [35]. The aging population is diverse genetically and is heterogeneous in terms of diseases and comorbidities. Because it is highly likely that the same mechanisms that drive aging also underlie multiple age-related chronic diseases, elucidation of these mechanisms may shed light on the aging process [36]. Model animals showing spontaneous onset of a pathology similar to that of a human disease have proven invaluable in this regard. Consequently, in the context of comparative studies on aging, there is a need for a greater number of diverse models of aging and age-related disorders in relation to any species of laboratory animals [37].

Such an approach to research can improve our understanding of the genes and their allelic variants that cause a disease early in life or predispose an individual to increased risk of a chronic disease. Thus, this approach may help to develop strategies to prevent or delay chronic medical conditions affecting the elderly. A crucial step in identifying the associations is defining the appropriate phenotype. Phenotypes of particular value for genetic research are those with high heritability and with close relations to gene products or pathways. A study involving congenic rat strains corresponding to disease states seems a sensible place to start.

As reported, two promising QTL locations on the 1^st^ chromosome of OXYS rats were potentially associated with the accelerated-senescence OXYS phenotype, including the effects on retinopathy, and at the same time, on some behavioral parameters (QTL1 is bounded by microsatellite markers D1Rat30 and D1Rat219) and on cataract, retinopathy, and some behavioral parameters together (QTL2 is in the vicinity of D1Rat219 and D1Rat81) [23]. We derived two congenic strains to verify the effects of each candidate locus: WAG/OXYS-1.1 and WAG/OXYS-1.2, respectively. The candidate genes were mapped within the certain congenic intervals on chromosome 1 by means of RNA sequencing (RNA-Seq) data [24]. We found that RNO1 segments that were introgressed from the OXYS parental strain spanned 81.1 Mbp in total (from position 8900000 to position 105000000 bp and from 178 000 000 to 275 000 000 bp, respectively) and identified clearly defined breaks in both regions. These congenic loci had effects on the development and progression of both cataract and retinopathy in both WAG/OXYS-1.1 WAG/OXYS-1.2 congenic strains. Such significant experimental data led to the assumption that the effects of the candidate genetic component may be dual: affecting the development of both ocular disorders. Thus, the present study was aimed at a thorough comparative analysis of the phenotypes of original congenic rat strains in order to compare the phenotypic effects of the proposed QTL locations and the actual congenic chromosome regions.

### Phenotypic characterization of WAG/OXYS-1.1 and WAG/OXYS-1.2 rat strains

In the present study, we assessed the progression of cataract and retinopathy with age in WAG/OXYS-1.1 and WAG/OXYS-1.2 rats through a series of successive ophthalmological examinations as compared to OXYS and WAG parental rats. We showed that the number of cataract-affected and retinopathy-affected congenic animals was markedly increasing with age, but the severity of both diseases in congenic rats was lower than in OXYS rats even by 12 months of age. Moreover, the clinical signs of retinopathy differed in congenic and parental rats, pointing to a strong effect of the genetic background. The histological examination confirmed the conclusion and revealed the characteristic features of retinopathy development in congenic and OXYS strains. Both WAG/OXYS-1.1 and WAG/OXYS-1.2 strains differed from OXYS rats by the parameters of the inner retinal blood supply and the inflammatory background of retinopathy development as indicated by the migration of immunocompetent cells into the inner plexiform and ganglion cell retinal layers. Accordingly, we concluded that similar mechanisms may underlie disease development and progression with age in both congenic strains.

One possible cause of the observed differences (in the retinopathy progression between OXYS and congenic strains, including the observed remission of 1^st^-stage retinopathy in WAG/OXYS-1.1 rats by age 12 months) is the differences in the state of animal immune system. Experimental and clinical lines of evidence strongly indicate the pathogenic role of the immunological processes in AMD, including the data on recruitment of macrophages, complement activation, microglial activation, and accumulation within the macula area [28, 38]. Because AMD is not accompanied by an intense inflammatory reaction, it is possible that age-related dysregulation of reparative parainflammatory mechanisms in the eye leads to a low-grade chronic inflammatory response in the context of AMD pathology [39]. We have previously found a lack of a T-cell component of the immune system during rapid involution of the thymus in OXYS rats [40]. In support of this notion, we observed selective changes in the expression of genes related to the immune system and inflammatory responses in the retina of OXYS rats when compared with Wistar rats [18] and when compared with congenic rats [24]. These specific alterations at both the prodromal (20 days) and the advanced (18 months) stages of retinopathy may lead to a specific metabolic basis for development and progression of the disease, with some variation between the examined rat strains. Thus, the gene or genes within mapped congenic segments do have an effect on the accelerated-senescence phenotype in OXYS rats, revealing some overlap because they cause early cataract and retinopathy in both WAG/OXYS-1.1 and WAG/OXYS-1.2 rat strains.

The level of significance of statistical parameters used for QTL mapping in the examined F_2_ population allowed us to suggest that QTL1 and QTL2 could affect some behavioral parameters, including the time to enter the central area of the OF test and the number of grooming acts (for QTL2 only) in the EPM test. Thus, we conducted the behavioral testing, as well as MRI in order to determine the presence of neurodegenerative changes in the brain of congenic rats.

Age-related neurodegenerative disorders, including AD, are characterized by a progressive cognitive decline and memory dysfunction. Our recent results offer promising evidence of the relevance of the OXYS rat model to the neurodegenerative processes that are observed in AD. The profile of AD-like pathology in OXYS rats is characterized particularly by the manifestation of behavioral alterations and learning and memory deficits as early as by 3 months of age [41–43]. In contrast, the present study showed no considerable differences in the locomotor activity, levels of anxiety, and learning ability between the parental WAG strain and congenic WAG/OXYS-1.1 and WAG/OXYS-1.2 strains. The results of PCA were as expected, given that a complex trait is analyzed and a large part of the congenic genome corresponds to the WAG strain.

Emerging evidence reveals widespread demyelination in aging and disease, but the nature of myelin pathology in the specific cases has not been well studied. Several studies have suggested, however, that certain structural MRI biomarkers possess some degree of diagnostic power in the differential diagnosis. Recent studies on animal models of AD revealed focal demyelination within amyloid-β plaques in the hippocampus [44]. MRI showed that the signs of neurodegenerative changes were present in OXYS rats at the age of 3 months and were well pronounced at the age of 12 months [13]. Nevertheless, we did not detect significant signs of demyelination in the brain of congenic rats in the present study. There was a substantially smaller number of demyelination lesions on T2 WI in the brain of congenic rats than in the brain of OXYS rats. The enlargement of the lateral ventricles, called ventriculomegaly, is associated with neurodegenerative diseases, such as AD, whereas the initial cause is often unknown. This state of affairs may be due to an imbalance between production and resorption of cerebrospinal fluid, typical for neurodegenerative diseases [29, 30]. On the other hand, when compared to OXYS rats, the specific area of the cerebral lateral ventricles was significantly larger in all three strains: WAG, WAG/OXYS-1.1, and WAG/OXYS-1.2. Thus, we likely are witnessing a specific characteristic of the WAG parental strain in congenic strains. According to our data, we believe that the observed enlargement of the lateral ventricles is not associated with behavioral abnormalities or memory deficits in congenic rats.

We can reasonably assume that the gene/genes that have structural and/or functional defects with an effect on brain pathophysiological processes in OXYS rats, do not likely fall into the congenic RNO1 segments in WAG/OXYS-1.1 and WAG/OXYS-1.2 strains. According to our findings, the genetic factors affecting the development of early cataract and retinopathy do not have effects on the accelerated-senescence brain phenotype in OXYS rats, at least independently of the genetic background.

### A comparative analysis of QTL regions and congenic segments in WAG/OXYS-1.1 and WAG/OXYS-1.2 strains

Key unaddressed questions in biology are whether genes are physically clustered with respect to their known functions or phenotypic effects; these questions lead to the special interest in analysis of QTL regions. In the present study, we compared the list of genes located within the putative QTL1 and QTL2 loci [23], in the initially mapped congenic segments [24], and within known rat QTL loci strongly associated with retinopathy or behavioral abnormalities according to RGD. The enrichment analysis was used to extract candidate pathways or individual candidate genes that could correlate with the phenotypic outcome in at least two rat strains.

We found that the genes associated with the AD-presenilin pathway [Panther: P00004] and the AD-amyloid secretase pathway [Panther: P00003] did not fall into the RNO1 regions, which were introgressed into the congenic rat strains from the parental OXYS strain. Therefore, these genes may have effects on the signs of accelerated brain aging in OXYS rats, which are not manifested in congenic rats. The genes of the AD-presenilin pathway and AD-amyloid secretase pathway are known to be particularly relevant to the neurological phenotype and may contribute to the progression of most common age-associated neurodegenerative diseases such as Alzheimer’s and Parkinson’s diseases [45–47]. We have recently demonstrated that the pathogenic pathways enriched with DE genes in the retina of OXYS rats when compared to congenic rats may also affect AD-related pathways. Thus, existing data indicate that the development of the accelerated-senescence phenotype in OXYS rats has much in common with the development of AD [19]. Neurodegenerative changes observed in the OXYS retina could be an ocular manifestation of systemic disease processes. The results of the present study suggest that genes involved in ribonucleoprotein complex biogenesis may also be relevant for the accelerated-senescence brain phenotype in OXYS rats. These data are in full compliance with the reports that impairments in protein synthesis may be some of the earliest neurochemical alterations in AD and that the RNA splicing alterations associated with the ribonucleoprotein complex are adversely affected early in the development of AD [48, 49].

We found that the WAG/OXYS-1.2 congenic chromosomal segment delineated in our mapping experiment contains the *Fxn* gene, which is a strong candidate for an *Arrd2* locus associated with age-related retinal degeneration in rats as reported in the Rat Genome Database [50]. Nevertheless, *Fxn* does not seem to be a candidate gene associated with retinopathy development in the congenic rats and OXYS rats because we did not detect either nonsynonymous SNPs in the coding sequence or differential expression of *Fxn* in the retina of the congenic rats [24].

Three genes—*Asrgl1* (also known as *Gliap*, which encodes atypical mammalian type I cytosolic L-asparaginase), *Dhdh* (encoding dihydrodiol dehydrogenase), and *Gpha2* (encoding the α2 subunit of thyrostimulin)—were found to be differentially expressed in the retina of the congenic rats when compared to OXYS rats and do not fall into the congenic segments. Thus, our data point to a differential effect of the genetic background on the regulation of these genes. We can hypothesize functional mutations that take place in the corresponding regulatory sequences, but further studies are needed. The *Asrgl1*, *Dhdh*, and *Gpha2* genes are located within at least two QTLs that are reported in the Rat Genome Database and are associated with behavioral abnormalities. This observation indicates that these genes may have an effect on the behavioral phenotype of OXYS rats.

The level of *Asrgl1* mRNA was significantly higher in the retina of both congenic rats than in the retina of OXYS rats (adjp-value 0.0342 and 0.0000013 for WAG/OXYS-1.1 and WAG/OXYS-1.2 strains, respectively). The corresponding L-asparaginase protein is particularly abundant in the brain, where it was shown to be exclusively expressed in astrocytes and to be present in structures reminiscent of glial endfeet. These data suggest the involvement of ASRGL1 L-asparaginase in astroglial production of the neuroactive amino acid L-aspartate [51]. It was reported that a missense mutation in ASRGL1 leads to photoreceptor degeneration resulting in progressive vision loss [52]. The *Dhdh* gene is located in a genomic region associated with tameness and aggression, and the corresponding protein product is associated with the metabolism of xenobiotics by cytochrome P450 according to KEGG pathways [53]. The strongest expression differences between the brains of tame and aggressive rats were shown for the *Dhdh* gene. The *Gpha2* gene is encoding a glycoprotein hormone alpha 2 subunit of heterodimeric corticotroph-derived glycoprotein hormone also referred to as thyrostimulin. The results of immunohistochemical analysis suggest that thyrostimulin is widely distributed in the rat central nervous system [54] and likely performs a critical function in the activation of thyroid-stimulating hormone receptor [55].

Summarizing all the data, we believe in the existence of a complex genetic architecture underlying the accelerated-senescence phenotype of OXYS rats, while the genetic components driving neurodegenerative alterations in OXYS rats’ retina and brain can probably be dissected. Nevertheless, further research is needed to determine the actual role of the identified candidate genes in formation of the accelerated-senescence phenotype in OXYS rats.

## Conclusion

It is useful to suppose that formation of an integrated phenotype of aging can be achieved on the basis of multiple traits that may have weak correlations but share the underlying genetic architecture. This notion is based on the hypothesis that convergent results from multiple aging-related traits will point up the pleiotropic signals responsible for the overall rate of the aging process. In the present study, we carried out comprehensive phenotyping of two original congenic strains that develop early cataract and retinopathy. The results offer several putative correlations linking the candidate genes and pathways to the etiology of age-related neurodegenerative changes in OXYS rats’ retina and brain. On the basis of the above findings, the WAG/OXYS-1.1 and WAG/OXYS-1.2 congenic rat strains can be considered a valuable tool for further research aimed at the genetic dissection of the accelerated-senescence phenotype in OXYS rats and the relations among the candidate genes.

## Methods

### Animals

Male rats of the senescence-accelerated OXYS strain, WAG/OXYS-1.1 and WAG/OXYS-1.2 congenic strains, inbred Wistar Albino Glaxo (WAG) strain with a normal phenotype, and outbred Wistar stock with a normal phenotype were used in the study. All experimental animals were maintained by the Breeding Experimental Animal Laboratory of the Institute of Cytology and Genetics (SB RAS) as described previously [19]. The OXYS rat strain was derived from Wistar stock as described earlier [56]. The congenic rats show spontaneously developing cataract and retinopathy. The congenic rat strains were originally created at the Institute of Cytology and Genetics, (SB RAS), and the breeding scheme used to generate these congenic rats was published earlier [23]. Rats of the 9^th^ generation of WAG/OXYS-1.1 and WAG/OXYS-1.2 strains were used for phenotypic characterization in the study. Twenty-day-old congenic rats of the 11^th^ generation were previously used to obtain RNA-Seq data that are further analyzed in the study.

### Ophthalmoscopic examination

This examination was performed on 2-, 3-, and 12-month-aged rats of the parental (OXYS and WAG), and congenic (WAG/OXYS-1.1 and WAG/OXYS-1.2) strains after dilatation with 1% tropicamide using Beta Direct Ophthalmoscope (Germany) with a slit lamp. Assessment of the stages of cataract and retinopathy was carried out according to the Age-Related Eye Disease Study (AREDS) grade protocol [25] through the use of evaluation rating scales from 0 to 3 as described previously [23].

### Morphological and morphometric analyses of rat retinas

For the histological analysis, an AxioScop 2 Plus light microscope (Zeiss, Germany) was used. The study was carried out on OXYS, WAG, WAG/OXYS-1.1, and WAG/OXYS-1.2 rats aged 3 months (*n* = 5 per group), 10 months (*n* = 5 per group), and 16 months (*n* = 3 per group). These rats were ophthalmologically examined immediately before the tissue sampling and preparation. The eyeballs were immediately excised on ice, fixed in Carnoy’s fluid (alcohol: chloroform: glacial acetic acid at 6:3:1) during 2.5 h, and then washed thoroughly with 96% alcohol. Then the posterior wall of the eye was collected, fixed in 12% neutral formalin, carefully washed in water, dehydrated in ascending alcohol concentrations, and embedded in paraffin according to the standard technique. Serial semithin (4 to 5 μm thick) frontal sections were prepared on an LKB-4 ultratome (Sweden). After staining with hematoxylin and eosin (H&E) 5 random visual fields for each retina were examined using a Carl Zeiss Axiostar plus microscope (Germany). The frame area of 900 μm^2^ for the magnification of 10 × 100 was selected. The specific morphological parametres including areas of choroid vessels and the numbers of ganglion neurons were calculated separately using the Axiovision 4.8 software (Carl Zeiss Vision, Hallbergmoos, Germany).

### Behavioral testing

Behavioral testing started when the rats of OXYS, WAG, WAG/OXYS-1.1 and WAG/OXYS-1.2 experimental groups (*n* = 12 to 15 per group) reached the age of 3 months. Standard tests were conducted in the following order: the first one comprised observation of locomotor exploratory activity in an OF test at the age of 3 months [57]; the assessment of the degree of anxiety was conducted in the EPM test 1 week apart [58]; and then the same experimental animals underwent memory tests in the 8-arm radial maze at the age of 5 months [59]. The behavioral testing sessions were conducted between 8:00 a.m. and 12:00 p.m. because of a major circadian influence at least on the OF and EPM performance [60], and the testing order of the groups was counterbalanced.

### OF test

The apparatus consisted of an opaque plastic square (100 × 100 cm) surrounded by walls of 40 cm in height. A central area was arbitrarily defined as a square of 30 × 30 cm. A rat was assumed to be in the central area when its four paws were touching it. A central light source (100 W) on the ceiling provided invariant illumination in an otherwise dark room. Each rat was placed in the same corner of the maze facing the same direction, and its behavior was analyzed for 5 min. The following dependent variables were recorded: total distance moved, number of rearings, number of entries into the central area, time spent in the central area, number of grooming episodes, and the number of boluses.

### EPM test

The apparatus consisted of an opaque plastic central square platform (11 × 11 cm) with four arms (50 × 10 cm) forming a “plus” sign: two opposite arms without walls (open arms) and two opposite arms with 40-cm-high walls (closed arms). Each rat was facing an open arm and allowed to freely explore the maze for 5 min. The following dependent variables were recorded: total distance moved, the number of closed-arm entries, number and percentage of open-arm entries ([number of open arm entries × 100]/Total entries) and percentage of time spent in open arms ([time spent in open arms × 100]/300 s).

### Radial 8-Arm maze test

The apparatus consists of eight equally spaced arms (67 × 10 × 20 cm), made of grey Plexiglas and radiating from a circular central platform 26 cm across (Open Science, Russia). Each arm was equipped with a fully retractable guillotine door at its entrance and a magnetic food pellet tray at its end. Memory testing *per se* started after 2 days of habituation to the maze. According to the standard test variation, 2 trials per day with a 2-h interval between the trials were administered to each rat during 10 subsequent training days. An animal was placed on the central platform facing the same starting arm. After 20 s, all the doors were opened simultaneously, and rat’s behavior was analyzed for 15 min or until all four food-associated arms were visited. The food reward (dry cereal pellets, Lyubyatovo, Russia) was always placed in the same four arms of the maze. The following dependent variables were recorded: the total number of visited arms, the number of entries and time spent in arms with/without food reward, number of rearings. Memory errors were subdivided into reference memory (i.e., entering an arm that was never baited) and working memory errors (re-entering a previously baited arm during a trial). Working and reference memory performance was combined for four sessions for each animal: day 1 (a rat still getting acquainted with the maze), days 2–4, days 5–7, and days 8–10 of training, in order to control errors.

### MRI

These experiments on the brains of 12-month-old rats of parental (OXYS, WAG) and congenic (WAG/OXYS-1.1, WAG/OXYS-1.2) strains were performed on a horizontal 11.7 T magnet (Bruker, BioSpec 117/16 USR, Germany) operating at the ^1^H resonance frequency of 500 MHz. Eight rats per group were analyzed. Rats were anesthetized by injection of thiopental sodium (60 mg/kg intraperitoneally), during the procedures. The animal was placed in the prone position on an animal bed which was then slid into the magnet bore. A respiratory pillow placed underneath the lower torso was used to monitor respiration (SA Instruments, Stony Brook, NY, USA). The Multi Slice, Multispin Echo (MSME) technique was used. T2-weighted images (T2 WI) were obtained with the following scanning parameters: the repetition time (TR), 4200 ms; echo time (TE), 55 ms; matrix (MTX), 256 × 256; slice thickness, 1 mm; distance between consecutive slices, 1.2 mm; number of slices, 9; field of view (FOV), 4.0 × 4.0 cm. The brain structures were identified using the Rat Brain Atlas of Paxinos & Watson [61]. The foci of demyelination were visualized as hyperintense areas on the T2-weighed images, the number of which was counted in all axial sections of the brain.

### Gene expression analysis

The sets of DE genes in the retina of 20-day-old OXYS, WAG/OXYS-1.1, and WAG/OXYS-1.2 rats were analyzed previously as described by Korbolina et al. [24]. Briefly, the RNA-seq data were obtained in the amount of 40 million raw short reads per sample (in triplicate for OXYS and WAG/OXYS-1.2 strains, one pooled sample from the WAG strain and one pooled sample from the WAG/OXYS-1.1 strain). The analysis pipeline started with raw data. The Cutadapt tool have been embedded to remove adapters [62]. The pre-processed high quality reads were mapped onto the Rnor_5.0 reference genome assembly with TopHat2 [63]. ENSEMBL gene annotation data were used for the data conversion. The resulting gene count tables were utilized by DESeq package [64]. In all cases, *P-*values were corrected to q*-*values using the Benjamini-Hochberg method. The genes surviving at q <0.01 were determined as differentially expressed.

### QTL tracking on rat chromosome 1

Here, we compared the lists of genes of two QTL locations on chromosome 1 of OXYS rats with the RGD data for the rat QTLs. Two putative QTLs affecting the accelerated-senescence phenotype of OXYS rats were determined using F2 hybrids bred by a reciprocal cross with the WAG strain in the regions of microsatellite markers D1Rat30 and D1Rat219 (QTL1) and D1Rat219 and D1Rat81 (QTL2). The significant logarithm (base 10) of odds (LOD scores > 3) were obtained for early cataract (QTL2), and retinopathy (QTL1 and QTL2). In addition, the LOD scores ranging from 2.5 to 3 were obtained for some behavioral parameters recorded in the OF and EPM tests, including the time spent in the central area in both tests, the number of rearings in the OF test, and the number of grooming acts in the EPM test. These data led to the assumption that QTL1 and QTL2 might affect anxiety and the exploratory activity rate—and therefore certain behavioral signs of brain neurodegeneration—in OXYS rats [23].

The following QTL queries were analyzed for RNO1 according to the data present in the Rat Genome Database: “anxiety,” “anxiety response,” “locomotor activity,” and “behaviour.” These QTL reports (Table 2) provide a phenotype description, mapping, strain information, and links to candidate genes, if determined.

**Table 2:** The Rat Genome Database report for the analyzed quantitative trait loci on RNO1

RGD ID	Locus	Name	Start	End	LOD	Trait
1354647	Despr8	Despair related QTL 8	1	24368159	–	locomotor activity trait (VT:0001392)
1354653	Despr9	Despair related QTL 8	185390068	230390068	–	locomotor activity trait (VT:0001392)
738028	Anxrr12	Anxiety related response QTL 12	141296374	186296374	4.90	locomotor activity trait (VT:0001392)
738022	Anxrr13	Anxiety related response QTL 13	91093646	136093646	4.60	locomotor activity trait (VT:0001392)
738006	Anxrr14	Anxiety related response QTL 14	141296374	186296374	4.00	locomotor activity trait (VT:0001392)
2313402	Anxrr24	Anxiety related response QTL 24	49147799	156446783	–	aggression-related behavior trait (VT:0015014)

Start/End: start and end positions of QTL on RNO1, respectively, bp; LOD: the corresponding logarithm of the odds ratio, if reported

### Functional enrichment analysis

The enrichment analysis was used to characterize the genes localized within a certain RNO1 chromosome segment and to extract candidate gene clusters and/or pathways that may be clinically meaningful against the criterion of GO annotation. We also looked for individual candidate genes with functions that could correlate with the phenotypic outcome.

We used the functional annotation tool available in the Database for Annotation, Visualization and Integrated Discovery (DAVID) [65]. Clustering and grouping of the colocalized genes into functionally related gene groups (or classes) were performed using the DAVID gene functional classification tool based on shared functional annotation. This approach switches the functional annotation analysis from a gene-centric analysis to a biological-module-centric analysis. The minimum gene number in a seeding group was set to 2, and the similarity threshold was set to the minimum similarity threshold of 0.3, as suggested by the DAVID consortium. All remaining parameters were kept at their default values.

### Statistical analysis

The data were subjected to 2-way ANOVA or repeated measures ANOVA in the statistical package Statistica 6.0 (StatSoft, Tulsa, USA). A Newman-Keuls *post hoc* test was applied to significant main effects and interactions in order to estimate the differences between particular sets of means, unless noted otherwise. One-way ANOVA was used for individual group comparisons. The significance level was set to 5%. All data are shown as the mean ± SEM.

## Additional files

Additional file 1: Contains the results for the parameters of the blood supply in the retina of 10-month-old rats (Additional file 1: Figure S1) and for the locomotor activity of 3-month-old rats in the open field (OF) test (Additional file 1: Figure S2) (DOC 91 kb)
Additional file 2: Contains the lists of RNO1 genes which are located within examined regions of rat chromosome 1 (Additional file 2: Tables S1, S2, S3, S4 and S5) (XLSX 349 kb)
Additional file 3: Contains DAVID results for the set of separately analyzed 1498 genes (XLSX 156 kb)
Additional file 4: Contains RNA-Seq results by DESeq package (XLSX 6600 kb)

## Abbreviations

AD: Alzheimer’s disease
AMD: Age-related macular degeneration
bp: Base pairs
DE: Differentially expressed
EPM test: Elevated plus-maze test
GO: Gene ontology
KEGG: Kyoto Encyclopedia of Genes and Genomes
LOD: LOD score, log10 likelihood ratio, comparing single-QTL model to the “no QTL anywhere” model
Mb: Megabase (10^6^ base pairs)
OF test: Open field test
QTL: Duantitative trait locus
RGD: Rat genome database
RNO1: Rat chromosome 1
SNP: Single-nucleotide polymorphism
