# Supplementary material for: Genes of susceptibility to early neurodegenerative changes in the rat retina and brain: analysis by means of congenic strains
Source: BMC Genet. 2016 Dec 22;17(Suppl 3):153. doi: 10.1186/s12863-016-0461-7 (PMC5249004; doi:10.1186/s12863-016-0461-7)

**Supplementary Figure 1. The specific area of inner retinal vessels (A); the specific area of opened choroid vessels (B) in the retina of 10-month-old rats (n=3-6).** The data are shown as mean ± SEM. Abbreviations: *P < 0.05 for differences between the corresponding congenic rat strain and OXYS strain, # P = 0.007 for differences between WAG/OXYS-1.1 and OXYS rat strains.

**
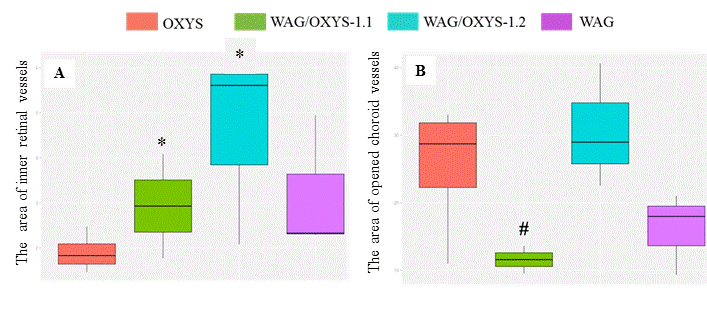
**

**Supplementary Figure 2. The locomotor activity of 3-month-old rats in the open field (OF) test.** The number of crossed squares was considerably reduced in OXYS rats as compared to age-matched WAG/OXYS-1.2 and WAG rats in the OF test. The data are shown as mean ± SEM. Abbreviations: *P < 0.05 for differences between the corresponding rat strain and OXYS strain.


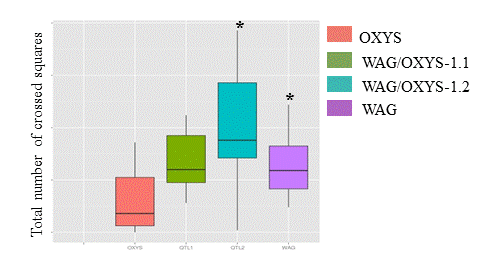

Supplement: Additional file 1: — Contains the results for the parameters of the blood supply in the retina of 10-month-old rats (Additional file 1: Figure S1) and for the locomotor activity of 3-month-old rats in the open field (OF) test (Additional file 1: Figure S2) (DOC 91 kb) [file 12863_2016_461_MOESM1_ESM.doc]
